# 572 Exploring Frailty in Rural Burn Patient Outcomes

**DOI:** 10.1093/jbcr/iraf019.201

**Published:** 2025-04-01

**Authors:** Mustafa Jundi, Alec McLeod, Garrett Hagwood, Alexa Smith, Jason Heard, Soman Sen, Tina Palmieri, Kathleen Romanowski

**Affiliations:** University of California Davis School of Medicine; University of South Dakota; Sutter Roseville Medical Center; University of California Davis Health; University of California Davis; University of California, Davis and Shriners Children’s; University of California, Davis and Shriners Children’s; University of California, Davis and Shriners Children’s

## Abstract

**Introduction:**

The Department of Agriculture estimates that 1 in 7 Americans live in rural areas. When patients from these regions sustain burn injuries, previous research shows they tend to experience larger burns and higher mortality rates compared to their urban counterparts. This demographic is often older, and experiences more chronic health conditions, factors often associated with frailty. Despite these findings, there is limited research on how frailty impacts burn outcomes among rural populations. This study aims to fill that gap by analyzing the effect of frailty on burn patient outcomes across these different communities.

**Methods:**

Following IRB approval, a retrospective chart review was conducted for burn patients over 50 admitted to a burn center between January 2021 and December 2022. Data collected included burn injury details, substance use, and patients’ reported zip codes. Rural-Urban Commuting Area (RUCA) codes determined by zip code were used to determine if patients lived in rural or urban areas. Frailty scores were calculated using the Canadian Study of Health and Aging Clinical Frailty Scale (CSHA CFS). Statistical analysis was conducted using SAS software (version 9.4) to perform Chi-square, Fisher Exact, and Wilcoxon 2-sample tests. Results are presented as median (interquartile range).

**Results:**

The study analyzed 451 patients with a median age of 62 (IQR 14). Of these, 305 (67.6%) were male, and the median burn size was 5% (IQR 11). A total of 46 (10.2%) died from their injuries. Among the participants, 110 patients (24.4%) resided in rural areas, and the median frailty score was 4 (IQR 2). Rural patients were more likely to be White (20.9% vs. 15.5%, p=0.005). No statistically significant differences were found between rural and urban patients in terms of age (63.5 years [IQR 16] vs. 62 [IQR 13], p=0.18), burn size (6% [IQR 12] vs. 5% [IQR 11], p=0.17), frailty (4 [IQR 1] vs. 4 [IQR 2], p=0.15), or mortality (9.1% vs. 10.6%, p=0.66). There were also no significant differences in length of hospital stay (13 days [IQR 19] vs. 10 days [IQR 18], p=0.37). There were also no differences in positive toxicology screen results (25.5% vs. 24.3%, p=0.19), methamphetamine positivity (24.6% vs. 23.3%, p=0.79), or alcohol use (6.4% vs. 6.1%, p=0.39) between rural and urban patients.

**Conclusions:**

This study found no significant burn characteristics or outcomes differences between rural and urban patients. Moreover, rural patients had similar rates of substance use compared to their urban counterparts. Based on these findings, healthcare providers should avoid making assumptions about a patient’s substance use or outcomes based solely on whether they come from a rural or urban area.

**Applicability of Research to Practice:**

Burn injury outcomes for patients from rural areas may be comparable to those from urban areas. This challenges previous assumptions and highlights the need for further research to address the specific needs of burn survivors, regardless of community type.

**Funding for the Study:**

N/A

